# Singlet Oxygen-Induced Mitochondrial Reset in Cancer: A Novel Approach for Ovarian Cancer Therapy

**DOI:** 10.3390/metabo14120648

**Published:** 2024-11-21

**Authors:** Jorgelindo da Veiga Moreira, Laurent Schwartz, Mario Jolicoeur

**Affiliations:** 1 Research Laboratory in Applied Metabolic Engineering, Department of Chemical Engineering, Polytechnique Montréal, Centre-Ville Station, P.O. Box 6079, Montréal, QC H3C 3A7, Canada; jorgelindo.daveiga@polymtl.ca; 2 Assistance Publique des Hôpitaux de Paris, Avenue Victoria, 75003 Paris, France; dr.laurentschwartz@gmail.com

**Keywords:** singlet oxygen, ovarian cancer, methylene blue, mitochondrial involution, metabolic bistability

## Abstract

**Background/Objectives**: This study explores the generation of singlet oxygen (SO) through methylene blue (MB) activation as a metabolic intervention for ovarian cancer. We aimed to examine the role of SO in modulating mitochondrial function, cellular metabolism, and proliferation in ovarian cancer cell lines compared to control cells. **Methods**: The study utilized two ovarian cancer cell lines, OV1369-R2 and TOV1369, along with ARPE-19 control cells. Following MB treatment and light activation, mitochondrial function and ATP synthesis were assessed. Metabolomic analyses were performed to evaluate changes in central carbon metabolism, particularly focusing on markers of the Warburg effect. **Results**: TOV1369 cells exhibited a pronounced sensitivity to MB treatment, resulting in significant inhibition of ATP synthesis and reduced proliferation. Metabolomic analysis indicated that MB-induced SO production partially reversed the Warburg effect, suggesting a shift from glycolysis to oxidative phosphorylation. These effects were less pronounced in OV1369-R2 and ARPE-19 cells, correlating with their lower MB sensitivity. **Conclusions**: MB-generated SO selectively modulates mitochondrial energetics in ovarian cancer cells, driving a metabolic reorganization that curtails their proliferative capacity. This approach, leveraging the bacterial-like features of cancer metabolism, offers a promising therapeutic avenue to induce apoptosis and enhance treatment outcomes in ovarian cancer.

## 1. Introduction

Ovarian cancer is still a challenging disease, and understanding its underlying mechanisms is crucial for developing effective therapeutic strategies. Metabolic reprogramming has emerged as a hallmark of cancer cells, providing them with a selective advantage for survival and growth [1,2,3]. A key aspect of cancer cells’ metabolic reprogramming relies on mitochondria dysfunction, thus affecting the cellular powerhouses responsible for energy production [4,5,6]. Mitochondrial alterations have been widely reported together with the promotion of glycolysis, a metabolic shift contributing to tumor progression and chemoresistance [7,8,9,10]. Indeed, targeting mitochondrial energetics has gained considerable attention as a promising strategy for inhibiting tumor growth and inducing cancer cell death [11,12,13]. Functional imaging techniques, such as positron emission tomography (PET scan), have revolutionized the detection and monitoring of cancerous tumors. PET scans—based on the injection of a fluorinated sugar, such as 18-fluoro-deoxy-glucose (18-FDG)—enable the visualization and assessment of cancer tissue based on enhanced glucose metabolism, evidenced by the accumulation of labeled glucose in the vicinity of the tumor mass [14]. This phenomenon of an increased energetic demand in tumors was initially elucidated by Otto Heinrich Warburg (1883–1970), a physician and Nobel laureate [15]. Warburg’s groundbreaking work revealed that cancer cells, even in the presence of oxygen, exhibit abnormally high lactate production compared to healthy cells, suggesting a shift toward glucose fermentation or aerobic glycolysis as the primary energy generation pathway in cancer cells. Warburg further hypothesized that the metabolic reprogramming observed in cancer cells might be attributed to mitochondrial dysfunction [16]. Subsequent studies, including independent research and validation from various groups, have contributed to the validation of several aspects of Warburg’s hypotheses. For instance, alterations in mitochondrial structure and function in cancer cells, including changes in oxidative phosphorylation and reactive oxygen species (ROS) production, have been demonstrated. These mitochondrial alterations are now known to result in a profound rerouting of cellular metabolism, primarily observed with the promotion of glycolysis, which provides a selective advantage for tumor growth and survival [17,18,19,20,21].

Evaluating the hypothesis on the reversibility of these altered mitochondrial functions has, indeed, recently guided the emergence of a new paradigm with a potential for new metabolic therapies [12]. Notably, investigations into photodynamic therapy (PDT) targeting mitochondria have demonstrated that the laser-induced generation of singlet oxygen molecules (^1^O_2_) stimulate mitochondrial genome replication in HeLa cells [22]. Moreover, high PDT-based singlet oxygen (SO) levels have been shown to induce extensive cell death in human breast cancer cells [23] and retinoblastoma cells [24]. The efficacy of PDT-mediated cell death is enhanced in the presence of the photosensitizer molecule methylene blue (MB), which is predominantly absorbed by mitochondria [24,25], thus preferentially favoring singlet oxygen generation in the mitochondrial volume. These findings have confirmed the potential of singlet oxygen as a means to modulate mitochondrial function while perturbing its bistability status, thus inducing programmed cell death in cancer cells. Moreover, recent research has supported the notion of metabolic reprogramming as an explanation for the acquired chemoresistance of cancer cells [1,20,26]. In the wake of these findings, the present study aimed to further characterize the potential of a MB-based metabolic therapy for cancer, focusing on ovarian cancer, which poses significant challenges to current treatment approaches, including chemotherapy. Furthermore, in a recent study, the efficacy of MB was demonstrated in vivo on ovarian tumors grafted onto mice [27]. By elucidating the underlying mechanisms in cancer cell metabolic rewiring by singlet oxygen, the present study also evaluates its potential for modulating cancer cells to death and, thus, can contribute to the development of effective therapeutic strategies for ovarian cancer and, potentially, other malignancies. Of global interest, this study may have broader implications for enhancing our understanding of the mitochondria system’s bistable behavior, balancing the metabolic flux towards cell energetics or promoting proliferative pathways. The understanding that emerges from this research, and which needs to be validated by further study, is the potential fundamental role of ATP synthase in the quantum tunneling of protons and electrons [28] and sustaining cancer cells’ malignancy [29].

## 2. Materials and Methods

### 2.1. Statistical Analysis

Data are presented as mean ± standard error of the mean (n = 3). Statistical analysis was performed with Excel^®^ and GraphPad^®^. Values of *p* ≤ 0.05 were considered significant and the notations of * (*p* ≤ 0.05), ** (*p* ≤ 0.01), and *** (*p* ≤ 0.001) were used to compare to the control group by Student’s *t*-test.

### 2.2. Cell Lines and Culture Medium

The epithelial ovarian cancer cell lines, OV1369-R2 (Cellosaurus ID: CVCL_9T12) and TOV1369 (Cellosaurus ID: CVCL_9T17), from a patient’s ascites or tumor [29], respectively, were used in this study with permission from the CHUM (Centre Hospitalier de l’Université de Montréal) Research Ethics Board (BD 04.002). The retinal epithelial cell line ARPE-19 was purchased from ATCC (Cederlane, CRL-2302, Burlington, ON, Canada) and used as a normal control. OV1369-R2 and TOV1369 showed resistance to carboplatin treatment in 2D cultures [30]. All three cell lines were cultured in complete OSE medium (Wisent, Cat. 316-030-CL, Saint-Jean-Baptiste, QC, Canada) and supplemented with 10% FBS (Wisent, Cat. 080-150, Saint-Jean-Baptiste, QC, Canada), 250 µg/mL Amphotericin B (Wisent, Cat. 450-105-QL, Saint-Jean-Baptiste, QC, Canada), and 50 mg/mL Gentamicin (Wisent, Cat. 450-135-XL, Saint-Jean-Baptiste, QC, Canada). For all experiments, cells were previously incubated at 37 °C, 21% O_2_ and 5% CO_2_.

### 2.3. Methylene Blue Treatments

Different concentrations (0, 0.1, 1, and 10 µM) of methylene blue (MB) (Laboratoire Chimie-plus, Saint-Paul-de-Varax, France) were tested on the three cell lines, with the objective of inducing a rapid response within a 24-h assay. The 10 µM concentration of MB (MB10) induced a 50% viability response in TOV1369, the most sensitive cell line tested. The MB10 concentration point was therefore applied for all experiments.

### 2.4. Laser Radiation and Singlet Oxygen Generation

A He–Ne laser at a wavelength of 630 nm was used for the excitation of methylene blue, promoting singlet oxygen production. The Ex/Em of ^1^O_2_ was 504/525 nm. Cells were grown on 24-well culture plates. Wells were irradiated at regular time intervals for a total of 15 J/cm^2^ in each condition. The Singlet Oxygen Sensor Green (SOSG) probe at an Ex/Em of 504/525 nm (ThermoFisher, Cat. S36002, Saint-Laurent, QC, Canada) was used as a highly selective marker of singlet oxygen production. Microplate Tecan Infinite M200 (REF. 30016056, Grödig, Austria), software i-control Version 1.9, was used to detect the total amount of SOSG in each cell culture.

### 2.5. Mitochondrial Membrane Potential and ROS Measurements

Mitochondrial membrane potential was assessed using the JC-1 probe (Invitrogen™, Cat. T3168, Saint-Laurent, QC, Canada), a cationic dye that accumulates in mitochondria in a potential-dependent manner. This accumulation results in a shift in fluorescence emission from green (~525 nm) to red (~590 nm), with a decrease in the red/green fluorescence ratio indicating mitochondrial depolarization, as specified by the manufacturer. Additionally, ROS levels were determined by separately detecting JC-1 monomers (green fluorescence) and J-aggregates (red fluorescence). The ROS index was calculated as the ratio of these fluorescent forms, reflecting the balance of oxidative stress. In brief, after cell treatment with methylene blue, followed by irradiation, cells were resuspended in the culture medium, washed twice with PBS, and incubated with JC-1 (10 µg/mL) at 37 °C for 10 min. Cells were then washed twice with PBS and resuspended in PBS for analysis by flow cytometry (FACS).

### 2.6. Flow Cytometry Analysis

The first cell viability tests were performed by an MTT assay kit (Sigma, Cat. 11465007001, Markham, ON, Canada) and then by flow cytometry (BD Biosciences, Canada) to bypass the calorimetric effects of MB. Viability was determined by considering the number of events normalized by Precision Count Beads™ (BioLegend, Cat. 424902, Cedarlane, Burlington, ON, Canada) in a cell suspension. Similarly, apoptotic cell populations were determined using Apotracker™ Green (BioLegend, Cat. 424902, Cedarlane, Burlington, ON, Canada) and normalized by the total cell population. For flow cytometric staining, 300 nM Apotracker™ Green per million cells was used in the 100 µL final PBS volume. The Alexa Fluor^®^ 488 channel (BD Biosciences, Mississauga, ON, Canada) was used for detection. All FACS data were analyzed using FlowJo™ v10.9 (BD Biosciences, Mississauga, ON, Canada).

### 2.7. ATP Assay

An ATP bioluminescent assay kit (Sigma, Cat. FLAA, Markham, ON, Canada) was used for the quantitative determination of the free cell ATP amount. A luciferin and luciferase assay cocktail were added to 1 × 10^6^ lysed cell extract; the light emitted was proportional to the intracellular ATP amount. The free ATP amount in each growth condition, and following methylene blue treatment, was determined based on the ATP standard curve.

### 2.8. Metabolites Measurement

The consumption of glucose, glutamine, and phosphate, along with the production of lactate and glutamate, was assessed by measuring their concentrations in cell culture media both before (OSE medium) and 24 h after treatment. Media samples were collected following treatment, and metabolite concentrations were analyzed using the Roche Cedex^®^ Bio Analyzer (REF: 06395554001, Laval, QC, Canada). Calibration standards for glucose (REF: 06343732001), glutamine (REF: 07395655001), glutamate (REF: 07395582001), phosphate (REF: 06990070001), and lactate (REF: 06343759001) were sourced from Roche and prepared in accordance with the manufacturer’s guidelines.

## 3. Results

### 3.1. Methylene Blue-Mediated Singlet Oxygen Generation

**Figure 1:**
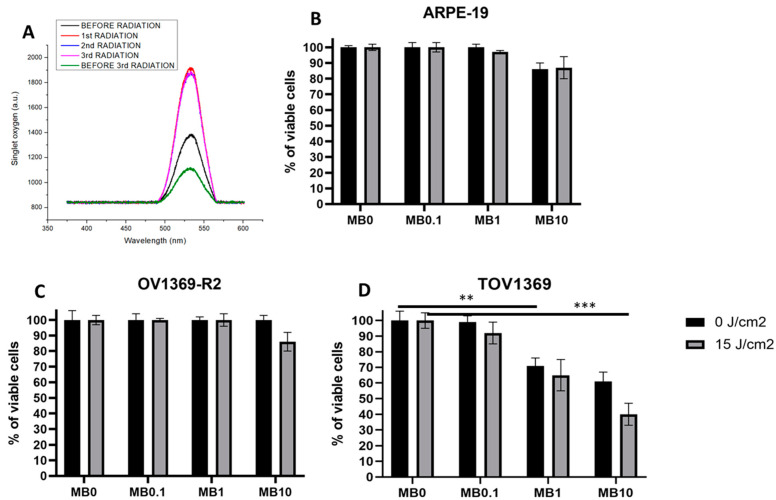
Methylene blue-induced mitochondrial singlet oxygen generation reduces ovarian cancer cell proliferation. (**A**) Singlet oxygen (SO) levels are monitored before and after laser radiation in cell cultures. Basal SO production (“before radiation”) is observed in all cell cultures in the presence of methylene blue (MB). Subsequent radiation rounds consistently result in the generation of SO to a level of 1900 a.u. (**B**) The effect of MB on ARPE-19 cell proliferation at a concentration of 10 µM (MB10) shows minimal impact, with no significant difference observed with the addition of SO. (**C**) The carboplatin-resistant ovarian cancer cell line OV1369-R2 displays limited sensitivity to low concentrations of MB. However, a slight inhibition in proliferation is observed at 10 µM of MB (MB10) when combined with SO. (**D**) The TOV1369 cell line, which is also carboplatin-resistant, exhibits a pronounced sensitivity to MB. The overflow of SO has a significantly greater effect on the culture condition with MB at a concentration of 10 µM (MB10). The *p*-values of ** (*p* ≤ 0.01) and *** (*p* ≤ 0.001) were considered as significant.

Methylene blue (MB) has demonstrated excellent photosensitizing properties and has been widely utilized for the generation of singlet oxygen (SO) in various cancer cell lines. In this study, we sought to investigate the capacity of MB to generate SO through successive radiation events, which was quantified using the SOSG reagent (Figure 1A). Our findings revealed that the presence of MB alone resulted in a basal level of SO production within the cell cultures. Upon exposure to a single radiation event, the basal SO production levels remained relatively unchanged. Interestingly, when subjecting the cells to successive rounds of radiation, we observed the consistent production of SO, reaching a steady state level of 1900 arbitrary units (a.u.) after a complete radiation dose of 15 J/cm^2^. These results highlight the robust and sustained generation of SO by MB, emphasizing its potential as an effective photosensitizer for targeted therapeutic applications.

### 3.2. Sensitivity of Ovarian Cancer Lines to Singlet Oxygen Overflow

To assess the impact of different methylene blue (MB) concentrations on ovarian cancer cell lines, we conducted experiments using various concentrations of MB (MB0 = 0 µM, MB0.1 = 0.1 µM, MB1 = 1 µM, and MB10 = 10 µM) and applied energy doses of 0 and 15 J/cm^2^. The effects of these treatments on two ovarian cancer cell lines (OV1369-R2 and TOV1369) (29) were compared to a normal epithelial cell line (ARPE-19). The results depicted in Figure 1B–D demonstrate that the combination of MB10 with a radiation dose of 15 J/cm^2^ exhibits a superior inhibitory effect on cell proliferation in both ovarian cancer cell lines. Notably, the TOV1369 cell line showed the highest sensitivity to this substantial production of singlet oxygen molecules, ^1^O_2_. Its sensitivity is dose-dependent, which is evident in response to MB alone as well as to ^1^O_2_. The most significant difference is observed between the MB alone condition and the condition of MB10 + 15 J/cm^2^. Under the latter condition, the production of ^1^O_2_ potentiates the inhibitory effect of MB on TOV1369 cell proliferation. In contrast, the OV1369-R2 line shows sensitivity to ^1^O_2_ only under the MB10 condition. Although this sensitivity is significantly higher than the MB10 condition alone, it remains considerably lower than the effect observed on the TOV1369 cell line (*p* ≤ 0.01 and *p* ≤ 0.01, respectively). Notably, MB10 alone or irradiated only slightly affected ARPE-19, which was to the same extent as OV1369-R2. These findings underscore the heightened sensitivity of some ovarian cancer cell lines to the overflow of ^1^O_2_, particularly when induced by the combination of MB10 and a radiation dose of 15 J/cm^2^. This approach could be used as a Trojan horse to control the central carbon metabolism in cancer cells, especially mitochondria-induced apoptotic activity.

### 3.3. Singlet Oxygen-Induced Apoptosis in Ovarian Cancer Cells

**Figure 2:**
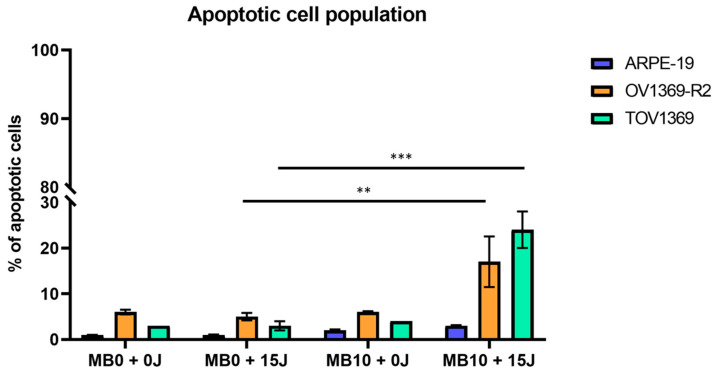
Singlet oxygen-induced (^1^O_2_) apoptosis in ovarian cancer cells. To assess the impact of singlet oxygen on ovarian cancer cells, we examined the occurrence of apoptosis under different radiation conditions using a 10 µM concentration of methylene blue (MB10) in a culture medium. The results demonstrate a significantly higher proportion of apoptotic cells in the OV1369-R2 and TOV1369 cancer cell lines compared to the normal ARPE-19 line (*p* ≤ 0.01 and *p* ≤ 0.001, respectively). This indicates the heightened sensitivity of the cancer cell lines to mitochondrial singlet oxygen generation. The observed increase in apoptotic cells supports the notion that ^1^O_2_ plays a crucial role in inducing programmed cell death in ovarian cancer cells. These findings underscore the potential therapeutic implications of targeting ^1^O_2_, a pivotal metabolite in the electron transfer chain (ETF), to selectively eliminate cancer cells while sparing normal cells. The *p*-values of ** (*p* ≤ 0.01) and *** (*p* ≤ 0.001) were considered as significant.

To elucidate the mechanisms underlying the reduction in proliferation observed in the ovarian cancer lines OV1369-R2 and TOV1369, we investigated the role of apoptosis as the primary pathway of cell death (Figure 2). Our findings reveal a significant induction of apoptosis in ovarian cancer cells exposed to singlet oxygen overflow. The extent of apoptosis is approximately three times higher in the OV1369-R2 line and around four times more pronounced in the TOV1369 line compared to other conditions. Interestingly, ^1^O_2_ induces specific apoptosis in cancer cells, as its effect is only marginally stronger in the normal ARPE-19 cell line. The observed induction of apoptosis in both ovarian cancer lines does not correlate directly with cell viability, suggesting the involvement of alternative mechanisms. In particular, the ability of OV1369-R2 cells to resist apoptosis may contribute to their reduced sensitivity to chemotherapeutic treatments compared to TOV1369. These findings highlight the significance of singlet oxygen-induced apoptosis in the ovarian cancer cell lines OV1369-R2 (*p* ≤ 0.01) and TOV1369 (*p* ≤ 0.001). They also emphasize the potential of targeting mitochondria for developing novel therapeutic strategies that consist of starving cancer cells by removing their non-optimal distribution of resources, such as through the use of fermentative bacteria.

### 3.4. Methylene Blue and Singlet Oxygen-Mediated Inhibition of Aerobic Glycolysis in Ovarian Cancer Cells

**Figure 3:**
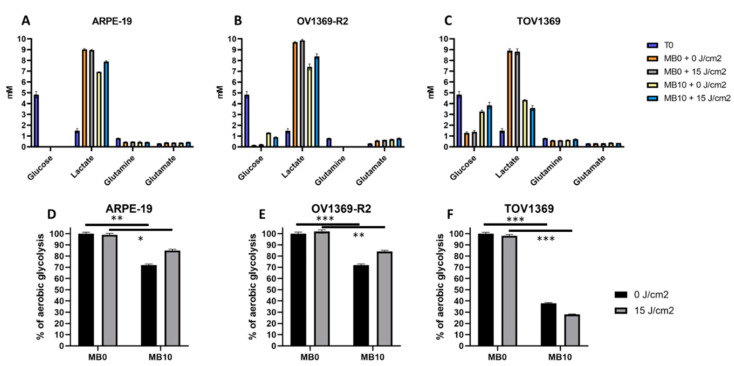
Methylene blue-induced singlet oxygen generation reduces aerobic glycolysis in ovarian cancer cells. (**A**,**D**) ARPE-19 exhibits a Warburg effect-like phenotype characterized by high glucose consumption and increased lactate production in the culture media. Treatment with MB10, with or without radiation, leads to reduced lactate production, indicating decreased aerobic glycolysis. (**B**,**C**,**E**,**F**) The MB10 treatment effectively attenuates lactate production in cancer cells, with a pronounced impact on TOV1369′s aerobic glycolysis. The OV1369-R2 line demonstrates higher glutamine consumption than the other two cell lines. The ”T0”condition refers to the initial metabolite’s concentration prior to cell incubation. The *p*-values of * (*p* ≤ 0.05), ** (*p* ≤ 0.01), and *** (*p* ≤ 0.001) were considered as significant.

The heightened anabolic demand of proliferative cells is primarily fueled by glucose and glutamine metabolism [31]. Under normal conditions, all three cell lines studied exhibit a substantial rate of glycolysis and citrate production (Figure 3A–C). In the absence of laser excitation, up to 9 g/L of lactate is produced (Figure 3B). Notably, the OV1369-R2 line demonstrates a higher rate of glutamine consumption compared to the other cell lines (Figure 3A,C). The presence of MB10 triggers glycolysis inhibition, specifically in the TOV1369 cell line (Figure 3C). This is accompanied by a notable reduction in lactate production, which is approximately halved compared to the control condition. The decrease in lactate production can be attributed to a clear reduction in glucose consumption. In the presence of singlet oxygen, the inhibition of lactate production and glucose consumption in TOV1369 is slightly augmented. In the OV1369-R2 cell line, aerobic glycolysis is also inhibited, but to a lesser extent compared to TOV1369. While lactate production is affected in ARPE-19, there is no significant impact on glucose consumption. Interestingly, the presence of singlet oxygen slightly elevates lactate levels compared to the MB10 condition alone. The observed reduction in aerobic glycolysis indicates a reversal of the Warburg effect, wherein cancer cells exhibit a preference for glucose and glutamine utilization for lactate production. To quantify the extent of the Warburg effect inhibition, the inhibition rate is calculated for all conditions relative to the normal condition (MB0 without laser excitation). As glycolysis contributes more significantly to the Warburg effect than glutaminolysis, the inhibition rate is higher in the TOV1369 line (Figure 3D–F). Under conditions of singlet oxygen overflow, the inhibition rate reaches 72%, while it is 62% under the MB10 treatment alone (Figure 3F). The OV1369-R2 line demonstrates a higher resistance to the effects of singlet oxygen on the Warburg effect. These results could provide information on the tissue specificity of mitochondria, which do not respond in the same way to energetic stimuli. This could be linked to the degrees of differentiation in cancer cells. This differentiation could be linked to mitochondrial energetic function.

### 3.5. Stimulation of Mitochondrial Membrane Potential and Restoration of Mitochondrial Energetics

**Figure 4:**
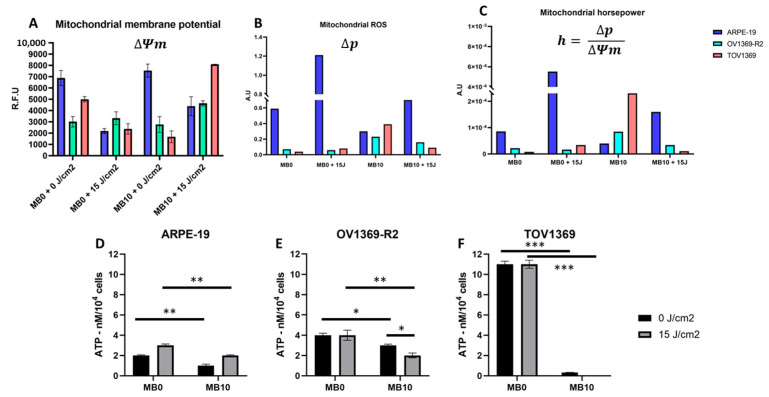
Singlet oxygen-mediated mitochondrial membrane potential stimulation rewires oxidative phosphorylation (OxPhos) in ovarian cancer cells. (**A**) Singlet oxygen increases the mitochondrial membrane potential (Δ*Ψm*), measured in relative fluorescence units (R.F.U) of the JC-1 cationic dye. (**B**) Mitochondrial ROS index (Δ*p*), determined by the JC-1 dye red/green fluorescence intensity ratio and measuring relative mitochondrial membrane polarization and depolarization, shows that the MB10 treatment increases mitochondrial ROS levels in cancer cell lines while having the opposite effect in the normal ARPE-19 cell line. (**C**) Mitochondrial horsepower (*h*), estimated based on ROS and MMP, is significantly increased in cancer cells under the MB10 conditions. With radiation, mitochondrial horsepower tends to approach values observed in the normal condition (MB0). (**D**–**F**) Free cellular ATP levels were assessed in the normal ARPE-19 cell line (**D**) as well as in the ovarian cancer cell lines OV1369-R2 and TOV1369. (**E**,**F**) In general, TOV1369 cells showed higher free ATP levels with a more pronounced response to MB10, indicating greater sensitivity. The *p*-values of * (*p* ≤ 0.05), ** (*p* ≤ 0.01), and *** (*p* ≤ 0.001) were considered significant.

The objective of these experiments was to assess the capacity of singlet oxygen (^1^O_2_) to stimulate the mitochondrial membrane potential (MMP) in cancer cells, aiming to counteract aerobic glycolysis. The results demonstrate a specific and significant stimulation of MMP in ovarian cancer cell lines (Figure 4). This stimulation is only observed under conditions of ^1^O_2_ generation (MB10 + 15 J/cm^2^). Notably, the ARPE-19 line, being a retina epithelial cell line, exhibits high sensitivity to laser excitation. However, the presence of MB10 appears to mitigate this effect on mitochondrial potential.

### 3.6. Revival of Oxidative Phosphorylation Reduces ATP Turnover Rate

Oxidative phosphorylation and glycolysis are the main pathways for ATP synthesis. The levels of free cellular ATP were quantified in the normal ARPE-19 cell line as well as in both ovarian cancer lines (OV1369-R2 and TOV1369). Overall, the amount of free cellular ATP was higher in the TOV1369 line (Figure 4D–F). These findings indicate a lower ATP turnover rate on methylene blue (MB10) in both cancer lines: OV1369-R2 (*p* ≤ 0.05) and TOV1369 (*p* ≤ 0.001). Notably, the effect is more pronounced in TOV1369, which is more susceptible to the presence of MB10. The amount of ATP decreases from 10.5 nM/10^4^ cells for MB0 without laser excitation to less than 1 nM/10^4^ cells treated with MB10. The presence of SO further limits ATP turnover rates in cancer cells compared to MB10 alone: OV1369-R2 (*p* ≤ 0.05) and TOV1369 (under the detection limit). The impact of SO is drastic in the TOV1369 line, significantly inhibiting the turnover of free cellular ATP (under the detection limit). These results highlight the direct influence of SO on rewiring the ATP synthesis pathway from aerobic glycolysis toward OxPhos, such as in normal differentiated cells.

## 4. Discussion

### 4.1. Rewiring Mitochondrial Energetics to Target Ovarian Cancer Cells

The findings of this study highlight the unique energy demand in proliferative ovarian cancer cells and their reliance on aerobic glycolysis for ATP production. The use of methylene blue as a Trojan horse, specifically targeting mitochondria in cancer cell cultures, led to a significant increase in singlet oxygen (SO) molecule, ^1^O_2_, generation and mitochondrial membrane potential. This suggests that singlet oxygen stimulates mitochondrial respiration and reactivates oxidative phosphorylation (OxPhos). Interestingly, despite the increase in mitochondrial potential, both cancer cell lines showed a drastic decrease in cellular ATP levels when exposed to SO (Figure 4). These results demonstrate the pivotal role of mitochondria in transitioning the central carbon metabolism and energy flows (Figure 3), providing a potential avenue for selectively limiting the proliferation of cancer cells.

### 4.2. Energy Starvation of Cancer Cells as a Strategy for Mitochondria-Directed Apoptosis

The impact of singlet oxygen on the viability of cancer cell lines appears to be closely tied to its effects on inhibiting the Warburg effect and stimulating OxPhos. The normal cell line ARPE-19 showed minimal effects on ATP production and apoptosis induction in the presence of singlet oxygen. In contrast, both cancer cell lines exhibited significant energy disruption, particularly TOV1369 (Figure 3). However, SO effectively induced apoptosis in OV1369-R2 cells, comparable to the response observed in TOV1369. These findings suggest that cancer cells adapt resource allocation to meet anabolic demand, primarily driven by ATP. Stimulation of mitochondrial activity serves to rectify this metabolic imbalance and restore basal metabolism. Notably, the normal cell line ARPE-19 tolerates the return to basal metabolism, while OV1369-R2 cells exhibit short-term resistance, and TOV1369 cells experience drastic inhibition (Figure 3 and Figure 4). Combining SO with chemotherapeutic treatments could potentially enhance the sensitivity of carboplatin-resistant ovarian cancer cells, providing a promising strategy for improving treatment outcomes [32].

### 4.3. Mitochondrial Horsepower: A Window into Modulating Mitochondria’s Cell Fate Decision

The concept of mitochondrial horsepower, represented by the equation *h* = Δp*(1/Δ*Ψ*) [33], offers insights into the functional modulation of the mitochondrial electron transfer chain (ETC) and its association with electrical power (*P* = U*I). The study demonstrates that exposure to MB10 results in an inverse relationship with the basal mitochondrial horsepower (*h*) for ARPE-19, OV1369-R2, and TOV1369 (Figure 4C). Under MB10 conditions, there is a significant surge in mitochondrial horsepower, especially for the TOV1369 cell line. This surge in the mitochondrial energetic mode, especially in cancer cells, is probably the result of a lower mitochondrial membrane potential (Δ*Ψm*) and the voltage across the mitochondrial dipole. Interestingly, laser irradiation and the subsequent generation of singlet oxygen appear to alleviate this surge, increasing the electric current through the cancer cell ETC and decreasing the mitochondrial horsepower (Figure 4A,C). Methylene blue’s ability to modulate the physico-chemical parameters of the ETC suggests the potential for controlling the cell fate decision—in this case, the apoptotic pathway (Figure 2). In summary, these results highlight the significance of mitochondrial rewiring, energy starvation, and the modulation of mitochondrial function as promising avenues for combating ovarian cancer (Figure 2, Figure 3 and Figure 4). The findings underscore the potential for personalized therapeutic approaches that leverage the unique metabolic characteristics of cancer cells (the Warburg effect and glutaminolysis) to induce controlled apoptosis and inhibit tumor proliferation (Figure 2 and Figure 3). Further exploration of these strategies holds great promise for improving the outcomes of ovarian cancer treatment and paving the way for innovative approaches to cancer therapy.

## 5. Conclusions

This study underscores the potential for harnessing the interplay between mitochondrial horsepower (*h*), singlet oxygen (SO), and methylene blue (MB) as a promising therapeutic strategy in the battle against ovarian cancer. By fine-tuning mitochondrial energetics and leveraging the metabolic susceptibilities of cancer cells, we have unearthed a novel path for targeted therapy. Through the ingenious use of MB as a Trojan horse, we have effectively directed ovarian cancer cell mitochondria energetics into a normal phenotype. This innovative approach yielded a profound surge in mitochondrial membrane potential, indicative of a revitalized mitochondrial energetic state closely mirroring that of normal cells. Mitochondrial horsepower, exemplified by the dynamic interplay between membrane potential (Δ*Ψ*) and proton-motive force (Δ*p*), has taken center stage as a pivotal determinant in the cell fate decision. Our findings elucidate the multifaceted impact of rewiring mitochondrial energetics through SO and MB interventions on ovarian cancer cells. Firstly, this intervention precipitates a significant reduction in aerobic glycolysis, the hallmarked metabolic pathway of cancer cells. This shift towards oxidative phosphorylation reinstates a more energy-efficient mode of ATP production, thus impeding mitochondrial replication and diminishing the proliferative capacity of cancer cells. Moreover, the selective activation of SO-induced mitochondrial modulation sets off a cascade of events, including ATP scarcity, culminating in controlled apoptosis. By capitalizing on the vulnerabilities of cancer cells and their reliance on a bacteria-like fermentative pathway, we have unveiled a means to induce targeted cell death while safeguarding normal cell viability. The concept of mitochondrial involution emerges as a potent framework for comprehending and manipulating cellular metabolism, paving the way for innovative treatment strategies across diverse cancer types, much like antibiotics revolutionized the fight against bacterial infections.

## Data Availability

The data underlying this study are not currently publicly available. The authors are currently assessing the possibility of patenting the findings and protocols presented in the manuscript.
